# Pharmacokinetics of Anti-HBV Polyoxometalate in Rats

**DOI:** 10.1371/journal.pone.0098292

**Published:** 2014-06-12

**Authors:** Juan Wang, Xiaofeng Qu, Yanfei Qi, Jinhua Li, Xiuling Song, Li Li, Dehui Yin, Kun Xu, Juan Li

**Affiliations:** School of Public Health, Jilin University, Changchun, Jilin, China; University of Illinois at Chicago, United States of America

## Abstract

Polyoxometalates are non-nucleoside analogs that have been proven to exhibit broad-spectrum antiviral activity. In particular, Cs_2_K_4_Na[SiW_9_Nb_3_O_40_].H_2_O 1 shows low toxicity and high activity against HBV. The preclinical pharmacokinetics of Compound 1 in rats were characterized by establishing and applying inductively coupled plasma-mass spectrometry method to determine the concentration of W in plasma, urine, feces, bile and organ samples. The quantitative ICP-MS method demonstrated good sensitivity and application in the pharmacokinetics study of polyoxometalates. The pharmacokinetic behavior of Compound 1 after intravenous or oral administration fit a two-compartment model. T_max_ ranges from 0.1 h to 3 h and the T_1/2_ of Compound 1 is between 20 h and 30 h. The absolute bioavailability of Compound 1 at 45, 180 and 720 mg/kg groups were 23.68%, 14.67% and 11.93%, respectively. The rates of plasma protein binding of Compound 1 at 9, 18 and 36 mg/ml of Compound 1 are 62.13±9.41%, 71.20±24.98% and 49.00±25.59%, respectively. Compound 1 was widely distributed throughout the body, and high levels of compound 1 were found in the kidney and liver. The level of Compound 1 in excretion was lower: 30% for urine, 0.28% for feces and 0.42% for bile, respectively. For elaborate pharmacokinetic characteristics to be fully understood, the metabolism of Compound 1 needs to be studied further.

## Introduction

Hepatitis B virus (HBV) infection, which is chronic and difficult to cure, clinical treatment, continues to be a major public health problem. In China, the hepatitis B virus surface antigen (HBsAg) carrier rate accounts for 9.8 percent of the population, and one third of the worldwide carrier [1]. HBV is the prototype of hepadnaviridae, a family of small, enveloped hepatotropic DNA viruses that can infect the liver of humans [2]. Infection by the virus results in a series of clinical symptoms ranging from minor flu-like symptoms to acute, fulminant or chronic hepatitis, liver cirrhosis, and liver carcinoma, and can even result in death [3]. HBV is estimated to be responsible for 500,000–700,000 deaths each year [4]. There are a number of drugs available to treat hepatitis B viral infection, including interferon (IFN), Thymosin α (Tα), famciclovir (FCV), adefovir (ADV), lamivudine (LAM) et al. However, none of these therapies are completely safe and effective, and clinical exploration of promising antiviral agents like nucleoside analogues is hampered by their significant side effects, especially the development of resistant viruses [5]–[7]. Therefore, it is crucial to explore the safer, more efficacious and less expensive anti-HBV agents.

Polyoxometalates (POMs) are inorganic cluster-like complexes that are constituted from the oxide anion and transition metal cations. These complexes are versatile and can be used in catalytic processes [8], magnetic materials[9], nanotechnology [10] and medical procedures. Several POMs are especially useful in medicinal chemistry, serving as new kinds of inorganic medicinal candidates that possess antiviral, antitumor, and antibiotic activities [11]–[14]. Because of their extremely small (sub 5 nm) dimensions, they exhibit low toxicity, are stable in biological media, are liable to be cleared by the renal system, and can be engineered for various applications, especially as antiviral agents [15]. POMs have been widely researched in recent years, and they have been proven to be active against a wide range of viruses, including both RNA viruses and DNA viruses, such as the human immunodeficiency RNA virus (HIV), severe acute respiratory syndrome RNA virus (SARS), influenza RNA virus and herpes simplex DNA virus (HSV) [16]–[19]. The mechanism of antiviral action may occur through the prevention of viral adsorption and penetration by inhibiting the activity of retroviridase [20]–[22]. The advantage of POMs in antiviral activity inspired us to find effective anti-HBV drug candidates in this field.

Among the various POMs, keggin-type niobium-substituted-heteropolytungstate Cs_2_K_4_Na[SiW_9_Nb_3_O_40_].H_2_O **1** interested us because of its broad-spectrum antiviral activity [23], especially for anti-HIV. Compound **1** has been synthesized, purified, and characterized, thereafter its toxicity and antiviral activity against hepatitis B virus were investigated in HepG 2.2.15 [24]. The results indicated that Compound **1** exhibits high activity against HBV and low toxicity.

Although POMs have been studied for many years, reports of relevant pharmacokinetics studies are relatively rare. There are, however, some papers from the 1990s [25], [26]. The most detailed published investigation of POM pharmacokinetics is that of Ni et al, and the atomic emission spectrometry methods for determination the concentration of POM in rats have already been described by this group. Since the limitations of the method, such as insensitive and less selective of detection limit, the inductively coupled plasma-mass spectrometry (ICP-MS) methods with more sensitively and selectively were first applied to determine the concentration of W in plasma, urine, feces, bile and organ samples. Findings from this study would be useful to evaluate the potential medicinal application of Compound **1**, and to perfect and complete the pharmacokinetics of POMs.

## Materials and Methods

### Drugs

Compound 1, which has been patented (Patent No. 201010192071.8), was synthesized in the School of Public Health, Jilin University, China, by using the procedures described in the literature[24]. Compound **1** was characterized by ultraviolet spectrometry, infrared spectrometry, and nuclear magnetic resonance. The molecular mass of Compound **1** is 3065 Daltons.

### Chemicals

The W standard solution (1000 µg/ml in 2% NaOH) was obtained from the National Analysis Center for Iron and Steel. Nitric acid (GR grade) was purchased from Damao Chemical Co. Ltd, (Tianjin, China). Ultra-pure water was prepared by passing deionized water through a Milli-Q water system (Millipore, Billerica, MA). In all cases, water was purified by deionization with a minimum conductivity value of 15 mΩ/cm. All other analytical grade chemicals were obtained from Chemical Co., China.

### Ethics Statement

Animals were maintained and experiments were conducted in accordance with the Institutional Animal Care and Use Committee, Jilin University, and with the recommendations in the Guide for the Care and Use of Laboratory Animals of the National Institutes of Health. The study was approved by the Animal Care and Use Committee of Jilin University (Permit Number: JLU2007–0003). All efforts were made to minimize suffering through the use of anesthesia, analgesia, and post-injury care and monitoring.

### Animals

Male and female healthy Wistar rats weighting 220 to 280 g were purchased from the Laboratory Animal Center of Jilin University, China. Rats were housed in a specific pathogen-free facility with controlled temperature of 24±1°C, a relative humidity of 55±5%, and a 12-h light/dark cycle (7:00 am to 7:00 pm). The animals were acclimated for one week in the Laboratory Animal Center of School of Public Health, Jilin University before any experimental procedures. All animals had free access to water and a standard rat diet. Food was withdrawn the night before the experiment; however, water was allowed ad libitum. Free access to food was resumed 4 h after dosing.

### Pharmacokinetic Study

#### Absorption Study

Oral administration: Rats were grouped randomly (three groups, n = 6 per group) based on their genders and body weights (BW). Compound **1** was orally administered to the three groups of rats at a single dose of 45, 180 or 720 mg/kg BW (5 mL/kg BW). Blood samples of 0.2 mL were collected into heparinized tubes at 0, 0.083, 0.25, 1.0, 2.0, 3.0, 4.0, 6.0, 12.0, 24.0, 48.0, 72.0 and 96.0 h via retrobulbar veniplex after the dose. The blood volume withdrawn at each time point was replaced with an equal volume of saline, administered orally, to compensate for fluid loss. Blood samples were immediately centrifuged at 3000 rpm for 5 min, and plasma was frozen at −20°C until analysis.

Intravenous administration: Six rats (three male and three female) were given Compound **1** at a single dose of 180 mg/kg BW (5 mL/kg BW) via caudal vein injection. Blood samples of 0.2 mL were collected into heparinized tubes at 0, 0.083, 0.25, 0.5, 1.0, 2.0, 3.0, 4.0, 6.0, 12.0, 24.0, 48.0, 72.0 and 96.0 h via retrobulbar veniplex after the dose. The blood volume withdrawn at each time point was replaced with an equal volume of saline, administered orally, to compensate for fluid loss. Blood samples were immediately centrifuged at 3000 rpm for 5 min, and plasma was frozen at −20°C until analysis.

#### Tissue distribution study

Wistar rats (*n* = 24, male and female in equal) were orally given Compound **1** at a single dose of 180 mg/kg BW. Five rats for each duration, 2, 24, 48 and 96 h post-dose, were sacrificed by decapitation after administration of Compound **1**. The whole brain, heart, liver, spleen, lung, kidney, uterus, testis, ovary, epididymis, stomach, intestine, pancreas, thymus, fat, and skeletal muscle were rapidly dissected and harvested. All tissues and organs were weighed on an analytical balance and frozen at −20°C until analysis.

#### Excretion study

Urine and feces excretion: Wistar rats (*n* = 6, male and female in equal) were orally given Compound **1** at a single dose of 180 mg/kg BW. Rats were placed in metabolism cages during the experiment to facilitate urine and feces collection. Urine and feces were collected during 0–1 h, 1–2, 2–4, 4–8, 8–12, 12–24, 24–48 and 60–72 h. Urine volume and feces weight were measured, and samples were frozen at −20°C until analysis.

Bile excretion: Rats (*n* = 6, male and female in equal) were anesthetized with diethyl ether, and bile duct were cannulated with a polyethylene tube. After recovery from anesthesia, rates were orally administered Compound **1** at a dose of 180 mg/kg BW. The bile samples were collected during 0–1, 1–2, 2–4, 4–8, 8–12, 12–24 and 24–48 h by bile duct cannulation. The bile volume was measured, and samples were frozen at −20°C until analysis.

#### Plasma Protein binding

Various amounts of Compound **1** were added to pooled male rat serum to yield concentrations of 9, 18 and 36 mg/mL. The dialysis membrane was Spectrapor II (Beijing Dingguo Changsheng Biotechnology Co. Ltd, China) with a molecular weight cutoff of 12,000 to 14,000. Preliminary studies demonstrated no adsorption of the compounds to the dialysis membrane. Serum (1 ml) was dialyzed against 10 mL of 0.1 mol/L isotonic sodium phosphate buffer (pH 7.4) in a shaking water bath at 37°C for 48 h, which was shown to be the necessary time for equilibration. Dialyzed serum and buffer volumes were collected and frozen at −20°C until analysis, and binding data were corrected for fluid shifts that occurred during dialysis [28]. Experiments were done in six parallel samples. The following formula was used to calculate plasma protein binding rate of Compound **1** under different concentrations:
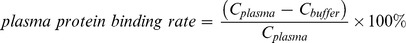



C_plasma_: concentration of Compound 1;

C_buffer_: concentration of Compound 1 in buffer

#### ICP-MS Conditions

A rapid inductively coupled plasma-mass spectrometry (ICP-MS) method was established to determine the concentration of W and Nb. An SCIEX ELAN DRC-e ICP-MS system (Perkin-Elmer Co., USA) was used. The instrumental parameters were as follows. The sample uptake rate was 1.0 ml/min, the plasma gas flow rate was 15 l/min, nebulizer gas flow rate was 0.89 l/min and the auxiliary gas flow rate was 1.20 l/min. The RF power was 1100 W, and the pulse stage voltage was 900 V. The scanning mode was peak hopping. Number of sweeps per reading was 3, the dwell time was 50 s, the sample flush time was 35 s, the sweep time was 20 s and the wash time was 45 s.

#### Sample Preparation

An aliquot of 0.1 mL plasma was transferred into a test tube, and 3 mL concentrated HNO_3_ was added. The test tube was laid on a hot plate, heated and refluxed at 120°C for 12 h until thorough dissolution. After cooling, the test tube was diluted with ultra pure water and made up to a final volume of 20 mL as analyte.

The urine, bile, postdialysis serum and buffer samples were aliquots into 0.1 mL, treated in the same way as plasma samples.

The tissue samples (except for fat and pancreas) were aliquots into 0.1 g and dipped in 3 mL concentrated HNO_3_ in test tubes. Each test tube was laid on a hot plate, heated and refluxed at 120°C for 12 h until thorough dissolution. After cooling, each test tube was diluted with ultra pure water and made up to a final volume of 20 mL as analyte.

Feces, fat and pancreas samples were aliquots into 0.1 g and dipped in 1 mL concentrated H_2_SO_4_ in test tubes. The test tube was laid on a hot plate, heated and refluxed at 60°C for 6 h. 3 mL concentrated HNO_3_was added at 120°C for 12 h until thorough dissolution. After cooling, the test tube was diluted with ultra pure water and made up to a final volume of 20 mL as analyte.

#### Assay Method

Concentrations of Compound **1** were determined by atomic spectrometry. Samples were analyzed directly by ICP-MS. Polyoxometalate concentrations were estimated directly from measured tungsten concentrations. Thus, this analytical method did not differentiate between parent compound and potential degradation or biotransformation products.

Quantization was based on the mean (*n* = 3) count of tungsten against a calibration curve by linear regression analysis, which was profiled with a series of standard tungsten solutions of different concentration.

#### Preparation of Standard solutions and Quality Control Samples

Stock standard solutions of the analyte (W) were prepared by separately dissolving the accurately weighed standard substances in NaOH and HF at a concentration of 10 µg/mL, and were stored at 4°C. A series of W standard working solutions (1, 5, 10, 50 and 100 ng/mL) were prepared by further dilution of the stock solution with an appropriate amount of ultra pure water. The working solutions were freshly prepared every week and stored at 4°C.

Quality control (QC) samples at low, medium and high concentration levels (5, 10 and 20 ng/mL) were prepared in the same manner. The same set of the QC samples were used for assessment of precision, accuracy, and recovery.

#### Method Validation

The limit of detection (LOD) and the limit of quantification (LOQ): The LOD and LOQ were determined by the lowest concentration to be detected as signals 3 times and 10 times higher than the baseline noise in blank sample, respectively.

The precision and accuracy of the method were evaluated using quality control (QC) samples in six replicates at three concentrations of 5, 10, and 20 ng/ml on 3 consecutive days, with two standard calibration curves for each analytical run. The precision and accuracy were expressed as relative standard deviation (RSD) and relative error (RE), respectively.

Recovery: Proper volumes of QC samples were spiked into blank plasma, tissues, urine, and feces to render solutions for testing with 5, 10, and 20 ng/mL. Experiments were done in six parallel samples, which were prepared, detected, and calculated by comparing the concentrations detected with the ones known in advance. Recovery was calculated as the formula: 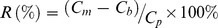, where C_m_ was concentration measured, C_b_ was blank concentration and C_p_ concentration prepared.

#### Data Analysis

The analytical method used for this study was not necessarily specific; rather, estimated polyoxometalate concentrations reflected concentrations of metal (W) atom. Thus, the pharmacokinetic analysis described the overall disposition of parent polyoxometalate and possibly degradation and metabolic products as well.

Pharmacokinetic analysis was performed using Kinetic 5.0 computer software (Thermo scientific Inc., USA). Results were expressed as the mean±standard derivation (SD). Data were subjected to a one-way analysis of variance (ANOVA) followed by the least significant difference (LSD) post-hoc test, and differences were considered statistically significant at *P*<0.05. Linear correlation was done to evaluate the relationship between doses and some parameters and differences were considered statistically significant at *P*<0.01. Statistical analyses were performed with the SPSS 12.0 software package (SPSS Inc., USA).

## Results

### Method validation

Specificity: ICP-MS uses a special interface technique to incorporate the inductively coupled plasma with the quadrupole mass spectrum, involving a high temperature (8000 K) ionization source and a sensitive, rapid scanning mass detector. Compared with traditional element analysis methods, such as graphite furnace atomic absorption spectrometry (GF-AAS) and inductively coupled plasma atomic emission spectrometry (ICP-AES), ICP-MS provides improved sensitivity and selectivity and is very suitable for pharmacokinetic studies. In addition, ICP-MS can satisfy all the detection requirements of inorganic element analysis and the limit of detection can achieve the level of sub ppt, and it also has the ability to determine many elements at the same time.

The LOD and LOQ: The concentration of W in the blank plasma sample was detected. The LOD and LOQ, indicators of the sensitivity of the assay, were found to be 0.002 and 0.008 ng/mL, respectively.

Standard curves and linear ranges: The calibration curves were all linear with regression correlation coefficients(r) >0.999. The curve equations and correlation coefficients of plasma, tissues, urine, feces and bile were as follows: plasma: y = 9218.01x+128.70, r = 0.99961; liver: y = 8201.12x+77.93, r = 0.99982; fat: y = 8055.85x+105.55, r = 0.99966; skeletal muscle: y = 7506.4x+18.06, r = 0.99999; urine: y = 8217.29x+29.57, r = 0.99997; feces: y = 10091x+36.14, r = 0.99997; bile: y = 13897.3x+51.74, r = 0.99997. The linear ranges were 0.05∼100 ng/mL.

The precision and accuracy: The intra-day and inter-day precision (expressed as

RSD%) and accuracy (expressed as RE%) of the assay are shown in Table 1. Both intra- and inter-day precisions and accuracies are within acceptable limits (i.e., less than 15% for RSD% and RE%) for low QC, medium QC and high QC samples (8). Overall, the intra-day accuracy and precision for the assay were in the ranges −8.861∼12.74% and 0.581∼5.198%, respectively. The corresponding values for the inter-day runs were similar, giving a precision of 0.860∼5.047%.

**Table 1:** Precision and accuracy for the determination of W in rat plasma, tissues, urine, feces and bile.

	Concentration (ng/ml)		Accuracy	Intra-day Precision	Inter-day Precision
	Added	Found	RE (%)	RSD (%)	RSD (%)
Plasma	5	5.046±0.095	0.920	1.961	1.873
	10	10.056±0.140	0.555	1.335	1.396
	20	19.940±0.171	−0.300	0.634	0.860
Liver	5	5.613±0.283	12.263	5.198	5.047
	10	9.901±0.314	−0.986	3.447	3.169
	20	18.264±0.533	−8.681	1.260	2.916
Fat	5	5.234±0.153	4.674	2.307	2.920
	10	10.223±0.161	2.234	1.290	1.575
	20	20.021±0.394	0.104	0.875	1.970
Muscle	5	5.227±0.061	5.538	1.171	1.163
	10	10.304±0.102	3.042	0.990	0.992
	20	20.234±0.211	1.172	0.852	1.041
Urine	5	5.273±0.169	5.461	2.358	3.205
	10	9.958±0.154	−0.420	1.157	1.550
	20	19.789±0.203	−1.056	1.037	1.024
Feces	5	5.638±0.189	12.740	3.295	3.353
	10	9.346±0.287	−6.543	2.380	3.070
	20	19.065±0.207	−4.676	1.050	1.087
Bile	5	5.251±0.084	5.011	1.408	1.600
	10	10.309±0.099	3.089	0.988	0.957
	20	20.082±0.182	0.409	0.581	0.904

Recovery: The results of recovery are shown in Table 2. The mean recoveries in rat plasma, tissues, urines, feces and bile for the low, middle and high QC samples were all approximately 100%, which demonstrates that the recovery is high.

**Table 2:** Recovery of W in rat plasma, tissues, urine, feces and bile.

	Concentration (ng/ml)			Recovery (%)
	Blank	Added	Founded	
		5	5.046±0.019	96.0±1.4
Plasma	0.246±0.060	10	10.078±0.109	98.3±1.2
		20	19.973±0.102	98.6±0.8
		5	5.613±0.084	93.3±1.6
Liver	0.649±0.007	10	9.901±0.085	92.5±0.9
		20	18.930±0.581	91.4±2.9
		5	5.256±0.096	96.1±2.9
Fat	0.453±0.046	10	10.242±0.133	97.9±1.8
		20	20.028±0.426	97.9±2.4
		5	5.277±0.023	94.6±0.9
Muscle	0.545±0.025	10	10.304±0.042	97.6±0.4
		20	20.234±0.160	98.4±0.7
		5	5.168±0.039	97.5±2.4
Urine	0.294±0.081	10	9.958±0.131	96.6±2.0
		20	19.850±0.017	97.8±0.4
		5	5.638±0.085	101.0±1.7
Feces	0.590±0.003	10	9.612±0.243	90.2±2.4
		20	19.065±0.103	92.4±0.5
		5	5.251±0.056	95.7±1.7
bile	0.468±0.052	10	10.309±0.026	98.4±0.8
		20	20.082±0.172	98.1±0.7

### Pharmacokinetic parameters

**Figure 1:**
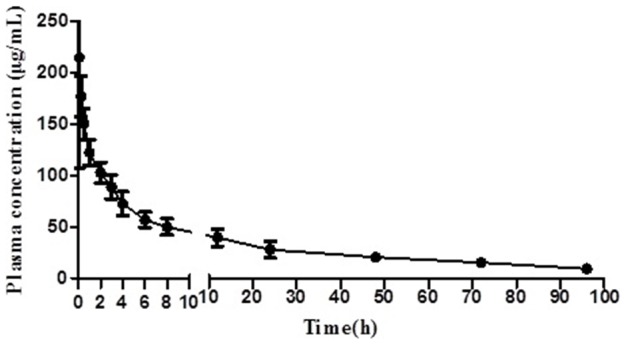
The plasma concentration-time profiles of Compound 1 after intravenous administration at 180 mg/kg Compound 1 in Wistar rats (mean ± S.D., n = 6).

The plasma concentration-time profiles of Compound 1 after intravenous administration were characterized in rats and illustrated in Figure 1. Data fitting was conducted and the results showed that the pharmacokinetic behavior of Compound 1 fitted a two compartment model. The main pharmacokinetic parameters of Compound 1 are summarized in Table 3.

**Table 3:** The main pharmacokinetic parameters of Compound 1 after intravenous administration at 180/kg Compound 1 in Wistar rats (mean ± S.D., n = 6).

Parameters	Units	Compound 1 injection
*t_1/2_*	h	30.76±4.658
*k_e_*	1/h	0.023±0.004
*T_max_*	h	0.139±0.086
*C_max_*	μg/mL	235.4±66.91
*AUC_0–96_*	μg •h/mL	2549±327.3
*AUC_0–_*_∞_	μg •h/mL	2965±342.8
*MRT*	h	47.05±3.529
*CL*	mL/h	15.34±1.695
*V_d_*	mL	679.7±114.7

t_1/2_, half-life; Ke: elimination rate constant; Tmax: time of peak concentration; Cmax: maximum concentration; AUC_0–96_: area under the curve up to 96 h; AUC_0–∞_, area under the total concentration-time curve; MRT, mean residence time; CL, systemic clearance; Vd, steady-state volume of distribution.

**Figure 2:**
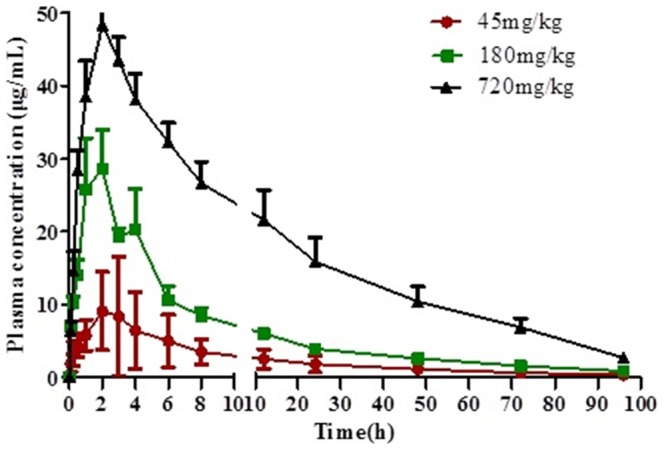
The plasma concentration-time profiles of Compound 1 after oral administration at 45, 180 and 720 mg/kg of Compound 1 in Wistar rats (mean ± S.D., n = 6).

Pharmacokinetic profiles of Compound 1 were investigated in Wistar rats after a single oral dose of 45, 180 or 720 mg/kg. The mean plasma concentration–time

curve of Compound 1 was presented in Fig. 2 and the pharmacokinetic parameters were listed in Table 4. Data fitting was conducted and the result showed that the pharmacokinetic behavior of Compound 1 fitted a two compartment model. Except for AUC_0–96_, AUC_0–∞_ and C_max_, no significant differences (P>0.05) were observed in the pharmacokinetic parameters among the three different groups. AUC_0–96_, AUC_0–∞_ and C_max_ are linear correlative with doses (P<0.01).

**Table 4:** The main pharmacokinetic parameters of Compound 1 after oral administration at 45, 180 and 720/kg of Compound 1 in Wistar rats (mean ± S.D., n = 6).

Parameters	Units	Dose(mg/kg)		
		45	180	720
*t_1/2_*	H	27.91±2.606	27.88±3.221	24.92±2.178
*k_e_*	1/h	0.025±0.002	0.025±0.003	0.028±0.003
*T_max_*	H	2.000±0.632	1.833±0.753	2.167±0.753
*C_max_*	μg/mL	11.13±8.370	30.85±14.25*	49.29±9.939*
*AUC_0–96_*	μg•h/mL	150.9±87.15	373.9±112.7*	1216±402.2*
*AUC_0–_*_∞_	μg h/mL	165.1±94.98	410.1±128.5*	1316±449.7*
*MRT*	H	36.45±4.495	35.42±5.940	37.57±4.676
*CL*	mL/h	86.96±42.45	117.1±29.49	153.7±62.35
*V_d_*	mL	3437±1593	4620±829.9	5583±2416

t_1/2_, half-life; Ke: elimination rate constant; Tmax: time of peak concentration; Cmax: maximum concentration; AUC_0–96_: area under the curve up to 96 h; AUC_0–∞_, area under the total concentration-time curve; MRT, mean residence time; CL, systemic clearance; Vd, steady-state volume of distribution. *P<0.05 among the three different groups.

The absolute bioavailability is the dose-corrected area under curve (AUC) orally divided by AUC intravenous. The formula for calculating F for a drug administered by the oral route (po) is given below.
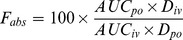



In our assay, the absolute bioavailabilities of Compound 1 at 45, 180 and 720 mg/kg groups were 23.68%, 14.67% and 11.93% respectively.

### Tissue Distribution

**Figure 3:**
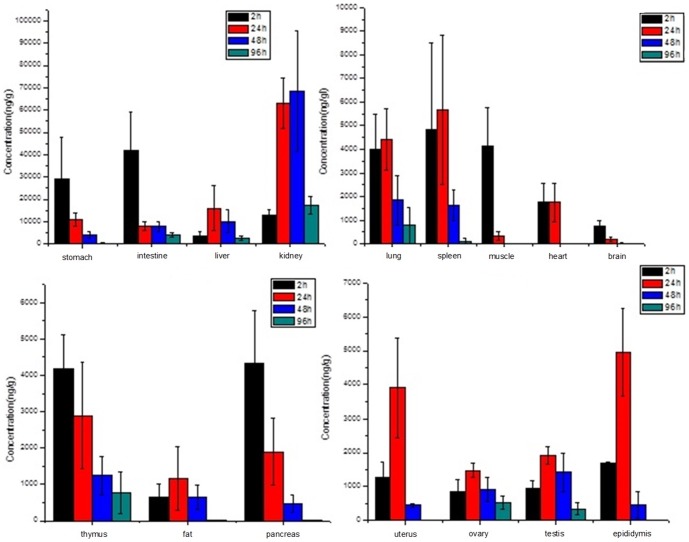
The schematic diagram of tissue distribution of Compound 1 in Wistar rats after 180/kg of orally administered Compound 1 (mean ± S.D., n = 6).

The tissue distribution of Compound 1 after oral administration at 180 mg/kg is presented in Figure 3. The highest deposition was found in the kidney, followed by the stomach, intestine and liver. Less deposition was found in the brain. These results show that the major organ for deposition of Compound 1 was the kidney.

### Excretion study

**Figure 4:**
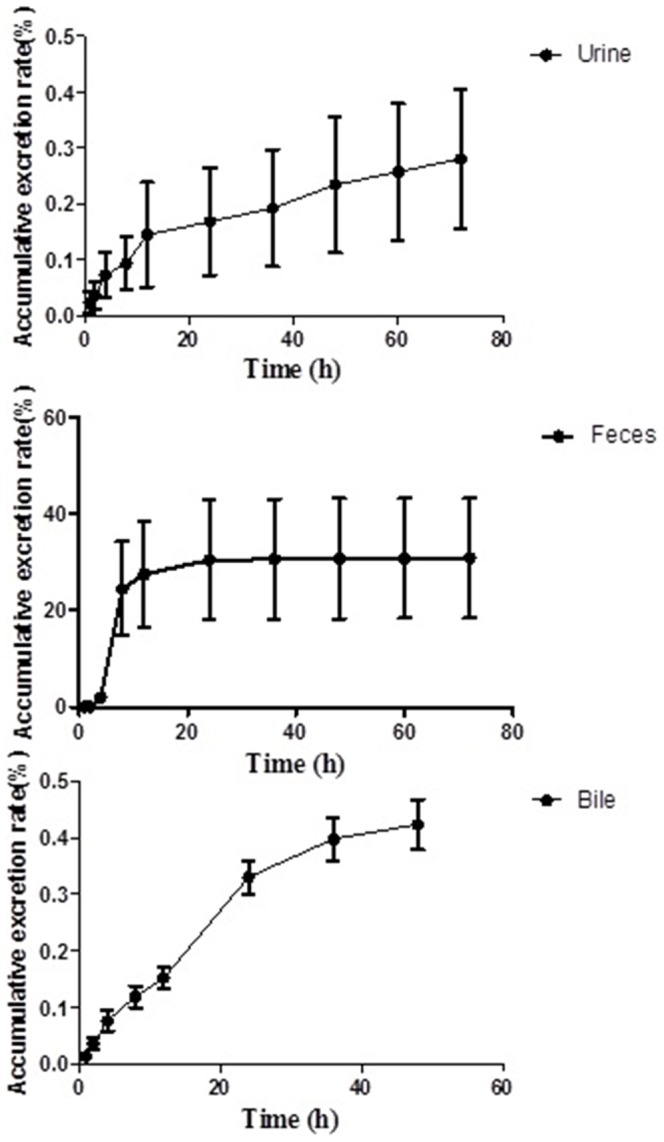
The schematic diagram of accumulative excretion rate of Compound 1 by urines, feces and biles after orally administered Compound 1 at 180/kg.

The accumulative excretion rate of urine, feces and bile are illustrated in Figure 4. The results indicated that the main excretion mechanism of Compound 1 was through feces, which accounted for about 30% of the drug tested, while urine accounted for approximately 0.28% and bile accounted for about 0.42%. Till 48 h, Compound 1 was excreted about 30.84% of the total amount of administrated, and till 72 h, Compound 1 was excreted about 31.1% of the total amount of administrated. The results also indicated that Compound 1 can be easily accumulated in vivo.

### Protein binding

The rats' plasma binding rates of Compound 1 are shown in Table 5. The percentages of Compound 1 bound at 9, 18 and 36 mg/ml are 62.13±9.41%, 71.20±24.98% and 49.00±25.59%, respectively, indicating that Compound 1 was highly bound to rat plasma protein at 9–18 mg/ml.

**Table 5:** Rat plasma protein binding rates at 48

Added Concentration (mg/mL)	Concentration in plasma (ng/mL)	Concemtration in buffer (ng/mL)	Protein binding rate (%)	Mean (%)	SD (%)
9	252621.45	131903.59	47.79	62.13	9.41
	85369.00	38664.01	54.71		
	529944.58	139267.14	73.72		
	206863.09	77194.24	62.68		
	363588.16	117217.22	67.76		
	363854.67	123223.95	66.13		
18	63951.73	6688.09	89.54	71.20	24.98
	501503.82	309160.02	38.35		
	625370.77	90086.12	85.59		
	2971773.88	134388.14	95.48		
	312035.10	72762.35	76.68		
	212941.68	124461.23	41.55		
36	747404.81	376223.58	49.66	49.00	25.59
	986379.93	482049.77	51.13		
	325246.21	233414.44	28.23		
	438586.00	329149.17	24.95		
	4278284.60	160165.98	96.26		
	574501.87	323047.84	43.77		

## Discussion

Treatment of patients with chronic HBV infection remains a critical clinical problem. Polyoxometalates, as non-nucleoside analogs, have been proven to exhibit broad inhibitory activity against HIV-1 and HIV-2, herpes simplex virus, influenza virus and SARS virus [27]. In our previous study, the treatment of HepG2.2.15 cells with Compound **1** effectively suppressed the secretion of HBV antigens and HBV DNA. [24] Therefore, the pharmacokinetics study of Compound **1** is critical to fighting viral atntigens.

The pharmacokinetics profiles of Compound 1 in rats were determined by quantitative ICP-MS method, which demonstrated good sensitivity, accuracy, precision, and recovery. The pharmacokinetic behavior of Compound 1 after intravenous and oral administration fitted a two compartment model. AUC_0–96_, AUC_0–∞_ and C_max_ are linear correlative with oral doses (*P*<0.01); however, t_1/2_, k_e_, CL, MRT and V_d_ do not change with the increase of administered doses, which indicates that the pharmacokinetic characteristics of Compound 1 comply with linear dynamics.

Compound 1 is able to be absorbed quickly into the blood circulation system after intravenous or oral administration to rats. T_max_ ranges from 0.1 h to 3 h and the half-life of Compound 1 is between 20 h to 30 h, which shows a good prospect of Compound 1 as a long-time-use drug for the treatment of chronic hepatitis.

In our assay, the absolute bioavailability of Compound 1 at 45, 180 and 720 mg/kg were 23.68%, 14.67% and 11.93% respectively, indicating that the oral bioavailability of Compound 1 in rats is low, and further study of absorption and elimination is also necessary.

After oral administration of 180 mg/kg of Compound 1, the compound is distributed to the 16 tested tissues. The highest deposition at 0.25 h was found in the stomach and intestine, but these concentrations decreased as time passed, which indicates that the main route of absorption is through the gastrointestinal tract. In addition to the gastrointestinal tract, Compound 1 also deposits in the liver, kidney, lungs and spleen, which have more blood flow. In contrast, Compound 1 deposits less in fat and skeletal muscle, which have little blood. The low concentration of Compound 1 in the testis, epididymis, ovary and uterus is consistent with the results in reproductive toxicity experiments, which found no obvious reproductive toxicity of Compound 1[28]. Much lower contents in the heart suggest that the cardiovascular system is not prone to being impaired by Compound 1. Less deposition was found in the brain, suggesting that Compound 1 cannot go through the blood brain barrier easily. The content of Compound 1 in the kidney and liver was as high as 10–70 times the content in the other tissues, which implied that kidney and liver were the main metabolism and elimination positions, and that they may the possible toxic sites. The high content in liver is also in favor of the treatment of chronic hepatitis. The clearance from tissues is rather quick; the content in tissues is less than 10% of the peak content at 96 h.

The percentages bound of Compound 1 at 9, 18 and 36 mg/ml are 62.13±9.41%, 71.20±24.98% and 49.00±25.59% respectively, indicating that Compound 1 was highly bound to rat plasma protein at 9–18 mg/ml and moderately bound at 36 mg/ml. The results indicate that most of Compound 1 exists in a combined form with plasma protein in systemic circulation; however, the binding force may be weak, and therefore it is likely to be out-competed by other high-binding drugs.

The accumulative excretion results indicated that the main route of excretion of Compound 1 was through feces, which contained about 30% of the drug tested, while urine contained about 0.28% and bile contained about 0.42%. At 72 h, approximately 31.1% of the total amount of administered Compound 1 had been excreted. The results also indicated that Compound 1 can be easily accumulated *in vivo*, so the further study of Compound 1 and metabolites from excretion is also essential.

In summary, a quantitative ICP-MS method was established and demonstrated good sensitivity and application in the pharmacokinetics study of polyoxometalates. The pharmacokinetic behavior of Compound 1 after intravenous or oral administration fitted a two compartment model. Compound 1 was highly bound to plasma proteins. Compound 1 was widely distributed throughout the body, and high levels of Compound 1 were found in the kidney and liver. The excretion of Compound 1 is lower in urine, feces and bile. Preclinical pharmacokinetics study shows that Compound 1 appears to have these above properties. These results could provide reference for further study of metabolism of compound 1.

In conclusion, we investigated the pharmacokinetics of compound 1 which possess inhibitory against HBV. Our data laid a good foundation for development of the POM as attractive anti-HBV drug.
